# N-Terminal Peptide of PGLYRP1/Tag7 Is a Novel Ligand for TREM-1 Receptor

**DOI:** 10.3390/ijms23105752

**Published:** 2022-05-20

**Authors:** Tatiana N. Sharapova, Olga K. Ivanova, Elena A. Romanova, Lidia P. Sashchenko, Denis V. Yashin

**Affiliations:** Laboratory of Molecular Immunogenetics of Cancer, Institute of Gene Biology RAS, 111394 Moscow, Russia; olga.k.ivanova@gmail.com (O.K.I.); elrom4@rambler.ru (E.A.R.); sashchenko@genebiology.ru (L.P.S.); yashin_co@mail.ru (D.V.Y.)

**Keywords:** TREM-1, peptides, PGLYRP1/PGRP-S/Tag7, cytotoxicity, cytokines

## Abstract

An investigation of innate immunity receptors sheds light on the mechanisms of inflammation and associated immune reactions. One of the key immune regulators is the TREM-1 receptor, which is involved in both inflammation and antitumor immune response. In this article, we have obtained a new ligand for the TREM-1 receptor. The peptide, named N3, is a part of the innate immune protein PGLYRP1/Tag7. It is responsible for activating the TREM-1 signaling pathway. Here, we have demonstrated that the N3 peptide acts like other TREM-1 receptor ligands: its binding results in a mild inflammation response and appearance of cytotoxic lymphocytes. We have shown that cytotoxic populations of lymphocytes in N3 peptide-treated PBMCs are similar to those treated with Tag7 or Hsp70. We also determined the part of the N3 peptide responsible for binding to TREM-1. The resulting peptide (N9) consists of nine amino acids and can be considered as a potential peptide that blocks TREM-1 signaling.

## 1. Introduction

All inflammation processes are based on recruitment and activation of innate immune cells. Pattern recognition receptors (PRRs) are the first line of defense against pathogens that can induce acute or chronic inflammation [1,2]. Among these, the family of Toll-like receptors (TLRs) plays a key role in innate immunity. They recognize structurally conserved molecules derived from bacteria and non-infectious antigens [3,4]. Activation of TLRs leads to hyperproduction of pro-inflammatory cytokines and downregulation of adaptive immune response [5,6,7].

Priming innate immune cells through TLR4 may also lead to upregulation and activation of another receptor of innate immunity—TREM-1 [8,9]. Activation of TLR4 triggers TREM-1 expression via myeloid differentiation primary response protein 88 (MyD88) and nuclear factor-κB (NFκB) [10,11,12,13]. Co-activation of TREM-1 through TLR4 amplifies the production of pro-inflammatory mediators [14,15]. In some pathologies, overstimulation of these receptors can be associated with sepsis, arthritis, colitis and infections [16,17]. In addition, TLR4-dependent activation of TREM-1 recruits matrix metalloproteinase (MMP-9), which is able to induce sTREM-1 release [18,19]. Detection of sTREM-1 is considered a biomarker for infection and viral disease, including COVID-19 [20,21,22].

In recent decades, TREM-1 ligands have been investigated as a potential tool to regulate inflammation. Recently, the following ligands of TREM-1 receptor have been identified: HMGBP1, PGLYRP1 and Hsp70 [14,23,24,25]. Some evidence suggests that Hsp70 is a novel ligand for TREM-1 and has an impact upon its activation [24,26]. The *PGLYRP1* gene in mice was first identified in IGB RAS [27,28]. It encodes a protein named Tag7. It has been demonstrated that Tag7 in combination with Hsp70 is able to induce TNFR-1-dependent programmed cell death [29,30]. On the other hand, Tag7 can be exposed on the membrane of CD4 + cells and recognize Hsp70 in tumor cells [31]. This stage is crucial in the elimination of tumor cells through FasL-Fas interaction by CD4 lymphocytes. It has been shown that incubation of Tag7 with PBMCs from healthy donors stimulates expression and release of pro-inflammatory cytokines, and also induces the appearance of cytotoxic lymphocytes that are able to eliminate HLA-negative tumor cells [32,33].

Our previous studies defined peptides of Tag7 with an ability to decrease inflammation in mice caused by acute lung injury (ALI) [34]. These peptides are located in opposite parts of the Tag7 protein and bind to different receptors. C-terminal peptides of Tag7 called 17.1 and 17.1A interact with TNFR-1, and the N-terminal peptide of Tag7, named N1, binds to the TREM-1 receptor. These peptides blocked signaling pathways from these receptors and reduced inflammation in both in vivo and in vitro experiments [34,35,36].

The aim of this study was to obtain a peptide that promotes an activation signal like the whole protein Tag7. To investigate functions of this peptide, we examined whether it can bind to TREM-1 and induce the receptor’s dimerization, followed by the determination of the expression of genes involved in the development of cytotoxic lymphocytes. Finally, we sought to define the shortest part of the peptide responsible for TREM-1 interaction.

## 2. Results

### 2.1. N-Terminal Peptide of Tag7 Is Responsible for Binding and Activation of TREM-1

**Figure 1:**
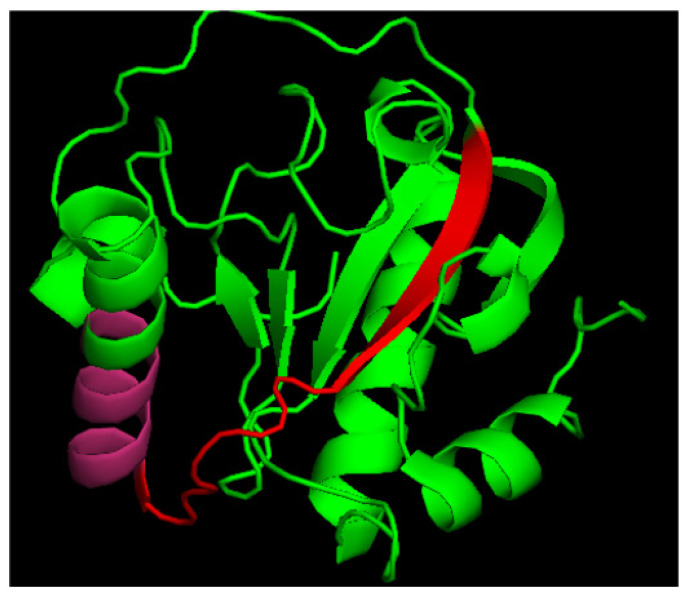
Crystal structure of human PGLYRP1. Red + pink—N3 peptide, pink—p.7 as a part of N3.

To obtain peptides of the protein Tag7, we used limited trypsin proteolysis. Samples after hydrolysis were separated by gel filtration on a Superdex peptide column. Then, we investigated the ability of the Tag7 fractions to induce cytotoxic activity in PBMCs. PBMCs treated by each of the fractions for 6 days were tested against K562 cells for cytotoxicity (Table 1). We detected that the fraction № 15 was able to demonstrate the same cytotoxic activity as Tag7-activated PBMCs. The sequence of this fraction was determined using MALDI TOF and corresponded to the N-terminal 24 amino acids domain RYVVVSHTAGSSCNTPASCQQQAR (Figure 1). This peptide was synthesized and named as N3. So, the N3 peptide reproduces the ability of Tag7 to induce cytotoxic populations of lymphocytes in PBMCs after 6 days of incubation.

**Figure 2:**
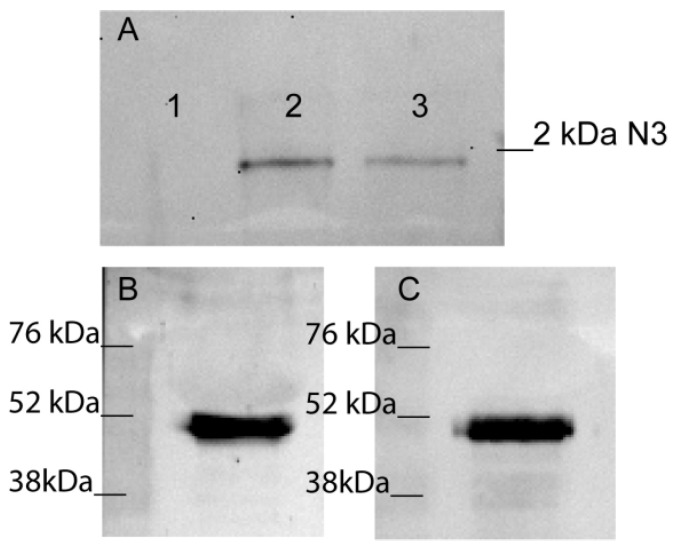
N3 peptide binds to TREM-1. (**A**) Elution of biotin-N3 peptide from sTREM-1 conjugated with CNBr-activated sepharose. 1—washing before elution, 2—elution of N3 peptide, 3—control N3 peptide. (**B**,**C**) Crosslinking N3 peptide with TREM-1 on monocytes surface. Binding N3 peptide with TREM-1 on monocytes surface in presence of BS_3_. Cells were lysed and purified with anti-TREM-1 Dynabeads (**B**) or anti-Tag7 Dynabeads (**C**). Bound material was analyzed by SDS-PAGE followed by WB and detected with anti-Tag7 (**B**) and anti-TREM-1 (**C**) respectively.

Based on these data, we suggested that N3 may reproduce the same ability to induce TREM-1 activation as the whole Tag7 protein. Our first goal was to demonstrate that the N3 peptide can bind to TREM-1. Using affinity chromatography, we found that the N3 peptide (biotin conjugated) binds to sTREM-1. Biotin N3 peptide was detected in elution material after tricine SDS-PAGE followed by streptavidin–HRP Western blotting (Figure 2A). Thus, we have demonstrated that the N3 peptide is able to interact with sTREM-1 in vitro.

The next step was to study the interaction of N3 and TREM-1 on the membranes of monocytes. The N3 peptide was added to PBMCs to bind with TREM-1 in the presence of a BS_3_-crosslinking agent. Cells were lysed and purified using anti-TREM-1 magnetic beads. The bound material was resolved by SDS-PAGE and WB and detected with anti-Tag7 antibodies (Figure 2B). The experiment was performed in reverse conditions with purification on anti-Tag7 magnetic beads and detection with anti-TREM-1 antibodies (Figure 2C). Both bands corresponded to a molecular weight of approximately 52kDA. This finding suggests that N3–TREM-1 interaction is associated with TREM-1 dimerization. It is well known that TREM-1 dimerization is the first step of signal transduction from the receptor [10,37]. Therefore, we have demonstrated that N3 peptide is able to bind to TREM-1 and induce dimerization of this receptor.

### 2.2. N3 Leads to Expression and Release Cytokines in PBMCs

**Figure 3:**
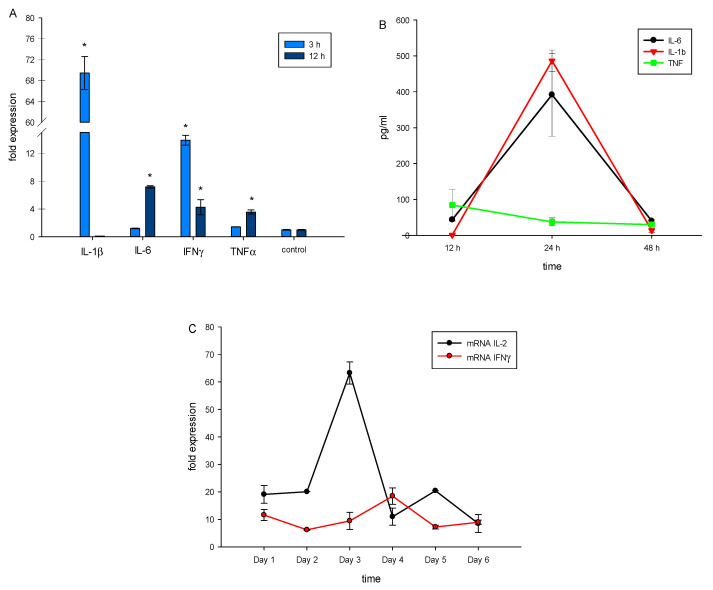
N3 peptide triggers mRNA expression of proinflammatory cytokines TNFα, IL-1β, IL-6 and IFNγ in PBMCs after 3 and 12 h of incubation (**A**). Secretion of proinflammatory cytokines by PBMCs after 12, 24 and 48 h of incubation with N3 measured using ELISA (**B**). mRNA expression of IL-2 and IFNγ, quantified using RT-PCR each day after N3 peptide treatment (**C**). All values were normalized to control (before any treatment). Levels of mRNA were measured by RT-PCR; levels of RPLP/GADPH were used as reference (one-way ANOVA, *p*-value * < 0.05 vs. control).

TREM-1 activation leads to expression of genes responsible for pro-inflammatory response. It results in the production of pro-inflammatory cytokines IL-1β, IL-6 and TNFα, as well as other cytokines that participate in maturation of cytotoxic lymphocytes (IL-2, IFNγ) [32]. Using RT-PCR, we demonstrated that in PBMCs treated with N3 mRNA, levels of pro-inflammatory cytokines TNFα, IL-1β, IL-6 and IFNγ increased after 3 or 12 h of incubation (Figure 3A). We also detected secretion of pro-inflammatory cytokines TNFα, IL-1β and IL-6 in a medium conditioned by N3-treated PBMCs (Figure 3B).

To track the influence of the N3 peptide on PBMCs during the entire incubation time, we measured gene expression of cytokines associated with maturation of cytotoxic lymphocytes—IL-2 and IFNγ. We observed high levels of IL-2 mRNA after 3 and 5 days of incubation, while mRNA of IFNγ achieved a maximum level after 4 days (Figure 3C).

Thus, interaction between the N3 peptide and TREM-1 initiates a sequence of events. Firstly, there is secretion of pro-inflammatory cytokines. After that, expression of IL-2 and IFNγ genes is activated. Appearing cytokines may induce the evolution of cytotoxic lymphocytes.

### 2.3. Incubation of N3 Peptide with PBMCs Reveals the Appearance of Cytotoxic Lymphocytes

**Figure 4:**
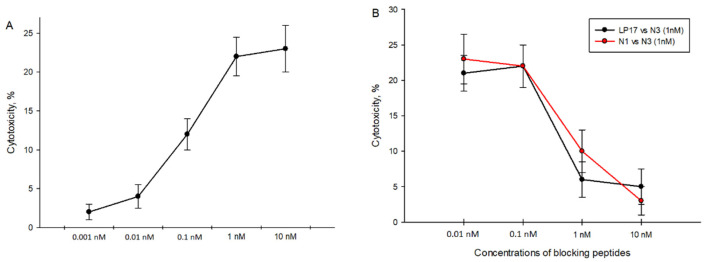
Cytotoxicity of N3-activated PBMCs against K562 cells. (**A**) N3-activated PBMC cytotoxicity depends on different concentrations of N3 peptides. (**B**) Cytotoxicity of N3-activated PBMCs in presence of TREM-1 inhibitory peptides: LP17 and N1. Concentration of N3 is constant in all cases—1 nM. PBMCs were treated for 6 days, cytotoxicity was measured after 24 h of incubation with K562 cells.

It was mentioned above that N3 stimulates the cytotoxic activity of lymphocytes. We found that maximum cytotoxicity of N3-activated PBMCs is achieved at 1 nM concentration (Figure 4A). We also tested peptides specific to TREM-1 to block transduction signals from the N3 peptide. PBMCs pretreated with various concentrations of LP17 and N1 peptides were incubated with the N3 peptide at a constant concentration of 1nM (Figure 4B). Cytotoxic activity during co-incubation of peptides decreased and was dose-dependent, where IC50 = 0.5 nM for both blocking peptides. Thus, the presence of blocking peptides LP17 and N1 during N3 treatment deteriorates the cytotoxic activity of lymphocytes. In this regard, we assume that N3–TREM-1 interaction is the first and most essential stage in the signal transduction pathway leading to the appearance of cytotoxic lymphocytes.

**Figure 5:**
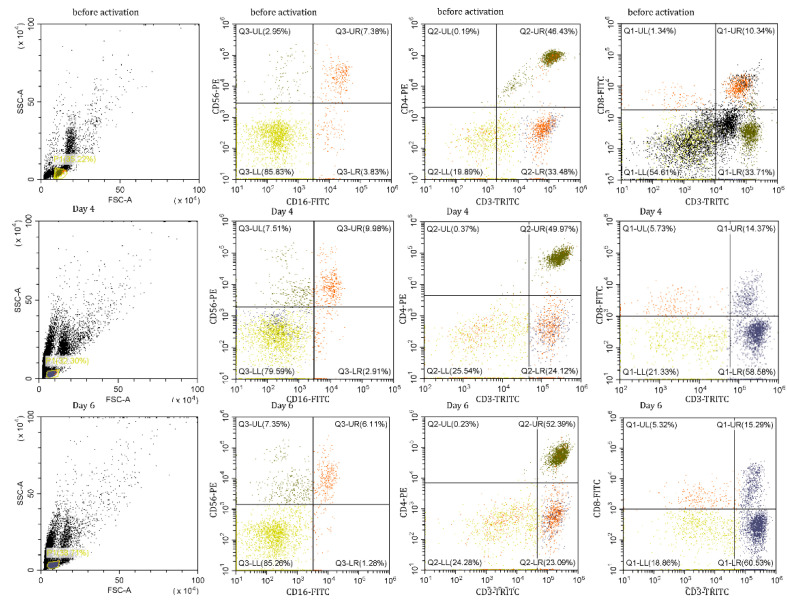
Ratios of lymphocyte subpopulations in PBMCs after N3 treatment.

As a next step, we have described cytotoxic subpopulations of PBMCs after N3 treatment. Using flow cytometry, we determined the pattern of CD16 + CD56 + CD3 + CD4 + and CD3 + CD8 + lymphocyte subpopulations, on day zero, day four, and day six of incubation with the N3 peptide (Figure 5).

**Figure 6:**
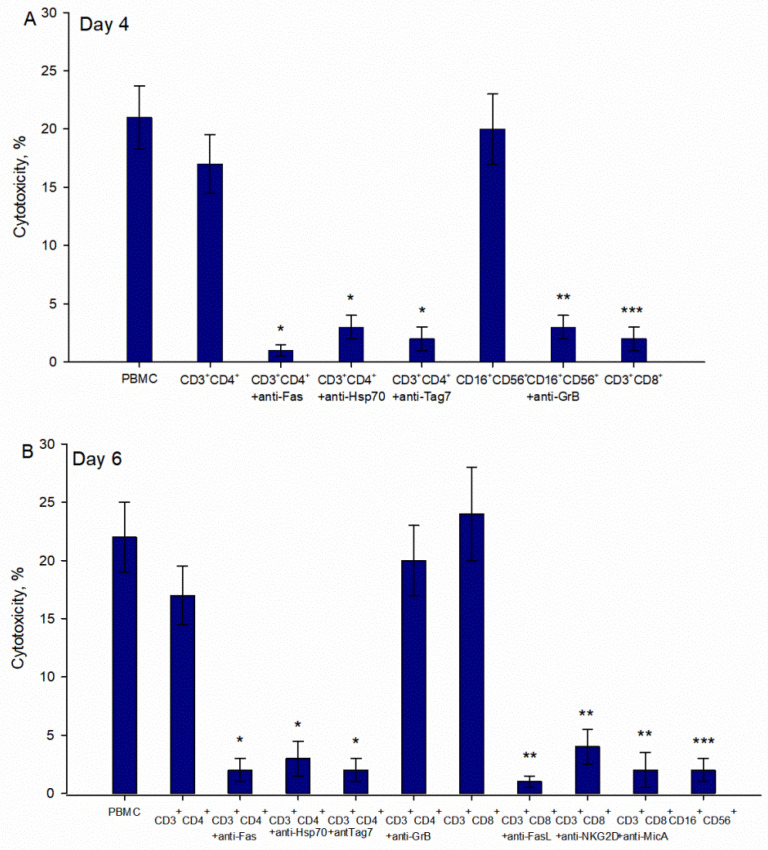
Cytotoxic activity of lymphocytes subpopulations after treatment of PBMCs with N3 peptide. Cytotoxicity of isolated CD16 + CD56 +, CD3 + CD4 +, CD3 + CD8 + lymphocytes from PBMCs incubated with N3 for 4 (**A**) and 6 (**B**) days. Subpopulations were isolated from N3 PBMCs using a Dynabeads negative isolation kit. Antibodies were added to target cells/lymphocytes before 1 h of cytotoxicity tests started. Cytotoxicity was measured after 24 h of incubation with K562 cells and after 3 h in tests with CD16 + CD56 + lymphocytes (one-way ANOVA, *p*-value: * < 0.05, *** < 0.05, ** < 0.03 vs. PBMCs).

It has been shown that the appearance of cytotoxic lymphocytes depends on the duration of incubation with the Tag7 protein [15]. The same results were obtained from our experiments with PBMCs treated with the N3 peptide. We detected cytotoxic populations on 4th and 6th days of treatment with the N3 peptide, and an absence of cytotoxicity on day 5 (Supplementary Materials Figure S1). We assumed that the phenotype of cytotoxic lymphocytes and the mechanism of their function should be the same as in the case of TREM-1 activation by other ligands. Our goal was to describe these cytotoxic subpopulations and the mechanism of their cytotoxicity. CD16 + CD56 + CD3 + CD4 + and CD3 + CD8 + were isolated from PBMCs treated with the N3 peptide on day 4 and 6 and tested for cytotoxic activity against K562 cells (Figure 6). Despite the presence of CD16 + CD56 + in PBMCs during the entire period of incubation, this subpopulation was able to induce granzyme-dependent cell death only on the 4th day of treatment. CD3 + CD4 + lymphocytes demonstrated their cytotoxic activity on both days. By using specific antibodies, it was proved that cell death was induced through FasL-Fas interaction and participation of recognition proteins—Tag7 and Hsp70—expressed on lymphocytes and tumor cell membranes, respectively. The data displayed in Figure 6 show that CD3 + CD4 + lymphocytes after 4 and 6 days of treatment used the same means to realize their cytotoxicity against tumor cells. The population of CD3 + CD8 + lymphocytes was able to demonstrate its cytotoxic activity after 6 days of treatment of PBMCs with N3. CD3 + CD8 + cells triggered cell death in target cells similarly to CD3 + CD4 + cells through FasL-Fas binding, but other recognition receptors, NKG2D and MicA, were used (Figure 6).

**Figure 7:**
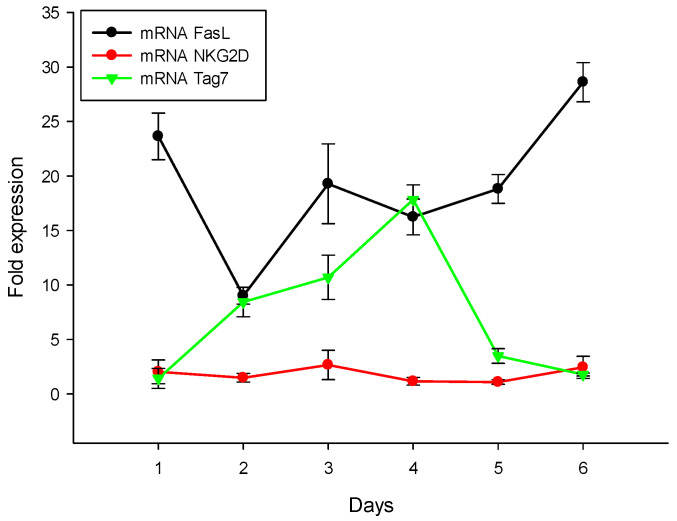
N3 peptide increases mRNA level of receptors of cytotoxic lymphocytes: FasL, NKG2D and Tag7 in PBMCs during 6 days of treatment. All values were normalized to control (before any treatment). Levels of mRNA were measured by RT-PCR. RPLP/GADPH were used as reference.

As mentioned above, the recognition of tumor cells by cytotoxic lymphocytes is a necessary stage to induce programmed cell death. This is why our next step was to track processes associated with the appearance of membrane proteins necessary for the recognition and elimination of tumor cells: FasL, Tag7 and NKG2D. We measured the mRNA profile of PBMCs treated with the N3 peptide and demonstrated that, from the first to the sixth days of treatment with the N3 peptide, the mRNA levels of FasL, NKG2D and Tag7 all increased (Figure 7). To summarize, N3 binds to TREM-1, and then dimerization of the receptor leads to signal transduction and appearance of the following cytokines: IL-1β, IL-6, TNFα, IFNγ and IL-2. At the same time, we observed mRNA expression of the proteins (FasL, NKG2D, Tag7) responsible for the induction of programmed cell death. It appears that these processes provide selective conditions for the appearance of the cytotoxic lymphocytes. Thus, the N3 peptide triggers TREM-1 dimerization, which sends an activation signal for the expression of certain cytokines, which in turn leads to a change in the repertoire of lymphocyte membrane proteins to induce programmed cell death.

### 2.4. 9-Mer Peptide of Tag7 Binds to TREM-1 and Prevents Development of Cytotoxic Activity of PBMCs

**Figure 8:**
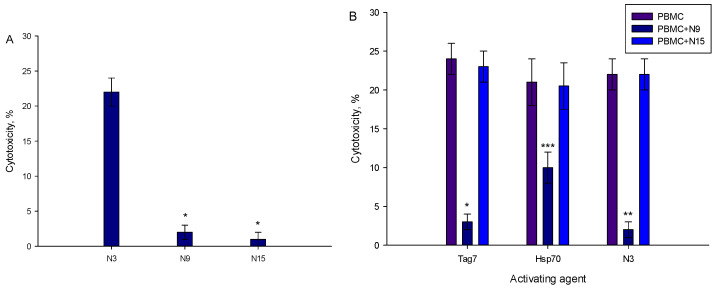

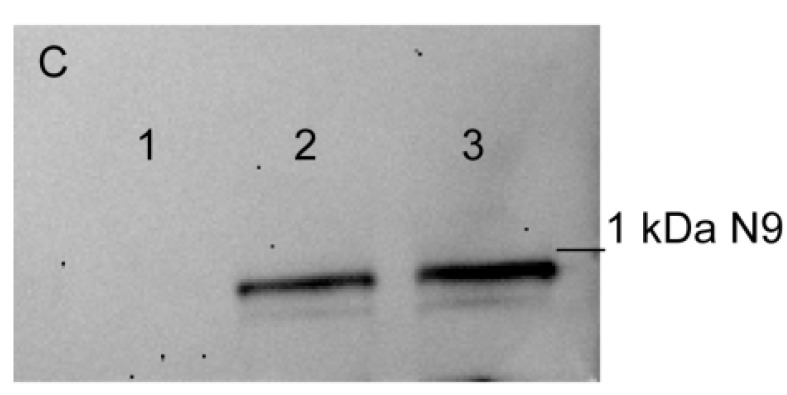
9-mer peptide of Tag7 binds to TREM-1 and prevents the development of cytotoxic activity of PBMCs. (**A**) Cytotoxicity of PBMCs treated with N3, N9 or N15 peptide. Concentration in all cases—1 nmol/L. (**B**) Influence of N9 and N15 peptides upon development of cytotoxic lymphocytes in PBMCs treated with Tag7, Hsp70 and N3 for 6 days. N9 or N15 was added to PBMCs before 1 h of activation with Tag7, Hsp70 or N3. Cytotoxicity was measured after 24 h of incubation with K562 cells (one-way ANOVA, *p*-value: *** < 0.05, ** < 0.03, *< 0.01 vs. PBMCs). (**C**) Elution of biotin-N9 peptide from sTREM-1 conjugated with CNBr-activated sepharose: 1—washing before elution, 2—elution of N9 peptide, 3—control N9 peptide.

As shown in Figure 1, the N3 peptide has two structural motifs: β-sheet and α-helix. Corresponding to these motifs, we synthesized peptides N15 and N9. Their sequences matched β-sheet (N15) RYVVVSHTAGSSCNT, and α-helix (N9) PASCQQQAR. PBMCs treated with each of the peptides demonstrated no cytotoxic activity against target cells (Figure 8A). The N9 peptide demonstrated an ability to block signal transduction from TREM-1 ligands: Tag7, Hsp70 and the N3 peptide (Figure 8B). There was no evidence for inhibitory activity of the N15 peptide (Figure 8B). Based on these data, we supposed that the N9 peptide should bind to the TREM-1 receptor. Using affinity chromatography, we detected that the N9 peptide interacts with sTREM-1 (Figure 8C). Therefore, the N9 peptide containing nine amino acids binds to TREM-1 and prevents its interaction with ligands.

## 3. Discussion

This study was based on the description of the functional part of the Tag7 protein, and as a result, two peptides were identified and synthesized. We have determined that the N3 peptide is responsible for binding to TREM-1 and activation of TREM-1 signaling, and thus reproduces Tag7 function. We have also identified and obtained another peptide, N9 (part of N3), and we suggest it should be considered as a novel TREM-1 blocking peptide.

The TREM-1 receptor is a key point of inflammation, as it acts like a chain in the signal amplification pathway initiated by the main pathogen recognition molecule TLR4. It is well known that activation of TLR4 triggers a release of pro-inflammation cytokines TNFa, IL-1b and IL-6, leading to an intense inflammation process [5]. There is some evidence that TREM-1 amplifies the inflammatory response initiated by TLR4, but TREM-1 ligation alone does not lead to sustained inflammation [10]. Common effects of TREM-1 and TLR4 receptor activation have also been described.

In this research, we discovered that stimulation of TREM-1 explicitly by the N3 peptide results in receptor dimerization and secretion of pro-inflammatory cytokines TNFα, IL-6 and IL-1β. Furthermore, the expression of IL-2 is induced. Switching the gene expression program from pro-inflammatory to activation cytokines may be the reason why sustained inflammation does not develop, but the formation of cytotoxic subpopulations of lymphocytes occurs. It was previously shown that IL-2 induces the appearance of cytotoxic lymphocytes in PBMCs, just as had been reported for Tag7 and Hsp70 [14,19]. TREM-1 ligand N3 peptides also promoted development of cytotoxic lymphocytes in PBMCs. They were able to recognize MHC-negative target cells by interaction with stress-induced molecules such as MicA and Hsp70. Their recognition mechanisms and cell death-inducing pathways were found to be similar in all cases. Thus, TREM-1 activation alone may lead to a slight pro-inflammatory response, and also to the development of a nonspecific immune response cause.

We have also described the function of the N9 peptide as a possible inhibitor of TREM-1. N9 is a part of the N3 peptide and is a sufficient motif to bind TREM-1. We have demonstrated in our previous works that the N-terminal peptide of Tag7 named N1 can bind to the TREM-1 receptor and decrease inflammation induced by LPS in PBMCs and mice [35]. Here, we have obtained the shortest peptide of N3 (N9) that is responsible for binding the TREM-1 receptor and does not reveal signal transduction. As well as other blocking peptides (LP17, N1), N9 can be considered a potential peptide capable of binding to the TREM-1 receptor and blocking its signaling pathway. Nevertheless, the role of the N15 peptide as a distinct part of N3 remains elusive. As a separate peptide, N15 did not demonstrate properties of an activating or blocking agent, but its function as a part of N3 remains a subject of investigation. Our data do not reveal the mechanisms behind the binding of TREM-1 with separate fragments of the N3 peptide. It is possible to speculate that the N15 motif is an essential part of N3, which stabilizes interactions between the ligand and receptor and promotes signal transduction. Exploring the function of N15 as a part of the N3 peptide in TREM-1 activation is a major limitation of this study that could be addressed in future research.

So, we have identified two peptides of Tag7, which can both be considered as novel ligands for the TREM-1 receptor. These peptides demonstrate opposite functions. The N3 peptide reproduces the function of the whole protein Tag7 and acts in a similar way. The N9 peptide is a part of Tag7 and is essential for binding to the TREM-1 receptor without signal transduction. These findings widen our understanding of mechanisms of innate inflammatory response regulation.

## 4. Materials and Methods

- Cell lines

Human peripheral blood mononuclear cells (PBMCs) were isolated from the total leukocyte pool of healthy donors as described in [29]. PBMCs and K562 cells were cultivated in RPMI-1640 with 2 mM L-glutamine and 10% FCS (Invitrogen, Carlsbad, CA, USA). Isolation of lymphocyte subpopulation from PBMCs was performed using monocytes NK, CD4 and CD8 negative isolation magnetic bead kits (Dynal Biotech ASA, Oslo, Norway) according to the manufacturer’s protocol. Cells were cultured with N3, Tag7, Hsp70, N15 or N9 at a density of 4 × 10^6^ cells/mL in the same medium.

- Proteins and Antibodies

Recombinant Tag7 and Hsp70 were prepared as described in [29]. The Pierce Chromogenic Endotoxin Quant Kit (Thermo Fisher Scientific, Waltham, MA, USA) detected no bacterial LPS in the recombinant protein preparation. sTREM-1 was obtained according to [37]. LP17 (LQVTDSGLYRCVIYHPP, 10^−9^ mol/L), N9 and N15 were added 1 h before lymphocyte treatment with activating agents. Anti-Hsp70, anti-Tag7 (anti-PGRP-S) (Thermo Fisher Scientific, Waltham, MA, USA), anti-Fas, anti-FasL (Santa Cruz Biotechnology, Santa Cruz, CA, USA), anti-Granzyme B (Abcam, Cambridge, UK) and anti-NKG2D (Santa Cruz Biotechnology, Santa Cruz, CA, USA) were added to cells 30 min before cytotoxic tests were performed.

- Peptides

Protein Tag7 was hydrolyzed at 37 °C for 5 h at a 1:10 trypsin/protein ratio (*w*/*w*) in 50 mmol/L (NH_3_)HCO_3_ (pH 8.0). The hydrolysate was then separated on a Superdex peptide column. Aliquots of each fraction (1 mL) were applied to the PBMCs. Peptides were synthesized as described in [36]. N9 and N15 were placed with Synpeptide Co., Ltd., (Shanghai, China).

- Mass Spectrometry

Matrix-assisted laser desorption/ionization mass spectrometry (MALDI) analysis was performed in the Institute of Biomedical Chemistry (IBMC). Data analysis was carried out using MASCOT^®^ peptide mass fingerprint software and NCBInr database (Japan).

- Affinity Chromatography, Immunoadsorption, and Immunoblotting

Two columns with CNBr-activated Sepharose 4B (GE Healthcare, Chicago, IL, USA) were pre-pared. Following the manufacturer’s protocol, one column was conjugated with sTREM-1. Biotin peptides were loaded onto the sTREM-1-Sepharose column. The columns were thoroughly washed with PBS containing 0.5 mol/L NaCl and PBS alone, and then eluted with 0.25 mol/L triethylamine, pH 12. The eluted material was resolved by Tricine SDS-PAGE, blotted onto a nitrocellulose membrane. HRP-conjugated streptavidin (GE Healthcare, Chicago, IL, USA; 1:15,000; 1 h) was used for detection. The results were visualized using ECL Plus Kit (GE Healthcare, Chicago, IL, USA) according to the manufacturer’s protocol. Chemiluminescence was detected using iBright (Thermo Fisher Scientific, Waltham, MA, USA). Monocytes (10^6^ cells) were incubated with N3 peptide (10^−8^ mol/L) in the presence of BS_3_ (Thermo Fisher Scientific, Waltham, MA, USA), lysed in RIPA buffer (SigmaAldrich, Burlington, CA, USA) and purified using Dynabeads (M-280 Sheep Anti-Rabbit IgG; PanMouse IgG; Dynal Biotech ASA, Oslo, Norway) conjugated with mouse anti-PGRP-S (Thermo Fisher Scientific, Waltham, MA, USA) or rabbit anti-TREM-1 antibodies (Abcam, Cambridge, UK) according to the manufacturer’s protocol. This material was resolved by 10% PAGE followed by Western blotting. To detect TREM-1, primary rabbit anti-TREM-1 antibodies (1:1000, overnight) or mouse anti-PGRP-S followed by secondary HRP-conjugated anti-rabbit or anti-mouse antibodies (GE Healthcare, Chicago, IL, USA; 1:15,000; 1 h) were used. The results were visualized using ECL Plus kit (GE Healthcare, Chicago, IL, USA) according to the manufacturer’s protocol.

- Cytotoxicity assays

K562 cells were cultured in 96-well plates (6 × 10^4^ cells per well), mixed with lymphocytes at a 20:1 ratio and incubated at 37 °C in a 5% CO_2_ atmosphere for 3 to 24 h. The inhibition test was conducted with polyclonal antibodies (anti-NKG2D, anti-MicA, anti-Hsp70, anti-Fas, and anti-granzyme B) at a concentration of 20 μg/mL. Cytotoxic activity of lymphocytes was detected with a Cytotox 96 Assay kit (Promega, Madison, WI, USA) according to the manufacturer’s protocol.

- Flow cytometry

PBMCs were stained with appropriate antibodies at room temperature. CD8-FITC and CD3 tri-color were obtained from Thermo Fisher Scientific (Waltham, MA, USA); CD4-PE, CD16-PE, CD56-FITC antibodies were obtained from Biolegend (San Diego, CA, USA). All antibodies were used in 1:200 ratio. The measurements were performed on a Cytoflex (Beckman coulter, Brea, CA, USA).

- ELISA

The levels of secretion of different cytokines were evaluated with Human IL-6, TNFα and IL-1β ELISA Kits (San Diego, CA, USA) according to the manufacturer’s protocols.

- RT-PCR

RNA was isolated from the fraction of PBMCs after treatment with the N3 peptide. PBMCs (4 mln) were lysed in 500 µL of TRI Reagent (Sigma-Aldrich, Burlington, CA, USA) according to the manufacturer’s protocol. RNA measurement was conducted with NanoDrop (Thermo scientific, Waltham, MA, USA) and equal amounts of RNA were used (1.1 µg). For detection of RNA, degradation electrophoresis was applied. The synthesis of cDNA was performed with oligo(dT) primers (Eurogen, Russia). The products were analyzed by qPCR with primers for genes encoding GAPDH, TNFα, IL-1β, IL-6, FasL, NKG2D, Tag7 and IFNγ. The levels of RPLP and GAPDH mRNA were taken as reference. The primers were as follows: for IFNγ: 5′ GGGTTCTCTTGGCTGTTACTG 3′ (forward), 5’ TTCTGTCACTCTCCTCTTTCCA 3′ (reverse); for TNFα forward: 5′-CTTCTCCTTCCTGATCGTGC-3′, reverse: 5′-GCTGGTTATCTCTCAGCTCCA-3′; FasL forward: 5′ GGATGTTTCAGCTCTTCCACC 3′, reverse: 5′ AGTTGGACTTGCCTGTTAAATGG 3′; NKG2D forward: 5′ AATTCCCTTGACCGAAAGTTACTG 3′, reverse: 5′ AGTAAATCCTGGTCCTCTTTGCT 3′; Tag7 forward: 5′ GCAACTACATGGATCGGGTG 3′, reverse: 5′ ATTCTGGATGAGGTGGTAGAGC 3′; IL-6: forward, 5′-GGAACAAGCCAGAGCTGTGC and reverse, 5′-TGCCGAAGAGCCCTCAGG; IL-1β: forward, 5′-GTACGATCACTGAACTGCACGC and reverse, 5′-CACGCAGGACAGGTACAGATTC; GAPDH forward: CAACAGCGACACCCACTCCT, reverse CACCCTGTTGCTGTAGCCAAA; RPLP forward: 5′-ACTGGAGACAAAGTGGGAGCC, reverse: 5′-CAGACACTGGCAACATTGCG.

Measurements at each point were made in at least three replications, and the mean value was calculated. Expression levels were quantified using the 2ΔΔCt method.

- Statistical Analysis

Data are presented as mean ± standard deviation. All experiments were repeated at least three times. Differences between treatment and control were tested for significance with SigmaPlot software (Systat Software Inc, Berkshire, UK) using one-way ANOVA (see individual figure legends).

## 5. Conclusions

In summary, we have identified two novel peptides of innate immune protein Tag7 able to bind to the TREM-1 receptor. One of them, the N3 peptide, acted similarly to other known ligands of TREM-1: Binding to TREM-1 caused its dimerization and the activation of genes responsible for development of cytotoxic lymphocytes. A part of the N3 peptide, N9, was also identified as a ligand for the TREM-1 receptor with the abilities of a blocking peptide. Further experimental investigations are needed to estimate the potential of using the described peptides as regulators of innate immune processes.

## Figures and Tables

**Table 1:** Cytotoxic activity of peptide fractions. PBMCs were treated with fractions for 6 days, cytotoxicity of lymphocytes was measured after 24 h of incubation with K562 cells.

Fractions	Cytotoxicity %	Fractions	Cytotoxicity %
1	2 ± 0.5	13	5 ± 1.7
2	3 ± 1.2	14	4 ± 1.4
3	5 ± 1.6	15	24 ± 3.1
4	2 ± 0.7	16	1 ± 0.6
5	4 ± 1.3	17	2 ± 1.2
6	1 ± 0.5	18	2 ± 1.3
7	6 ± 1.9	19	4 ± 1.8
8	5 ± 1.8	20	6 ± 2.1
9	2 ± 0.5	21	5 ± 2.0
10	2 ± 0.8	22	1 ± 0.6
11	3 ± 1.4	23	2 ± 0.5
12	2 ± 1.7	24	2 ± 0.6

## Supplementary Materials

The following supporting information can be downloaded at: https://www.mdpi.com/article/10.3390/ijms23105752/s1.
