# Supplementary material for: N-Terminal Peptide of PGLYRP1/Tag7 Is a Novel Ligand for TREM-1 Receptor
Source: Int J Mol Sci. 2022 May 20;23(10):5752. doi: 10.3390/ijms23105752 (PMC9144885; doi:10.3390/ijms23105752)
Supplement: Supplementary file 1 [file ijms-23-05752-s001.zip › ijms-1737157-supplementary.pdf]

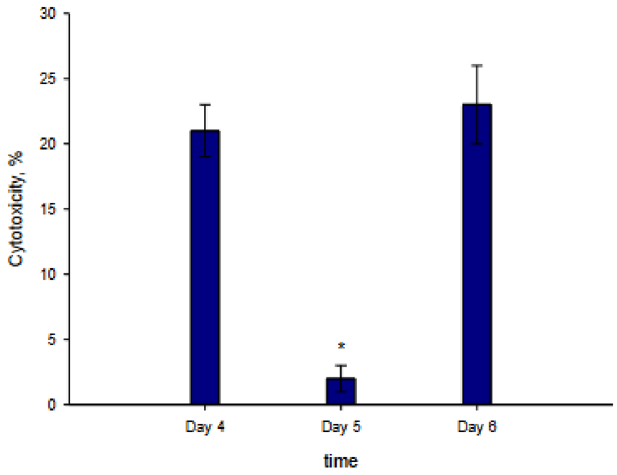


**Figure S1.** Cytotoxic activity of lymphocytes subpopulations after treatment of PBMC with N3-peptide. Cytotoxicity of PBMC incubated with N3 for 4, 5, and 6 days. Cytotoxicity was measured after 24 h of incubation with K562 cells (one-day ANOVA, p-value: * < 0.05 vs. PBMC).
